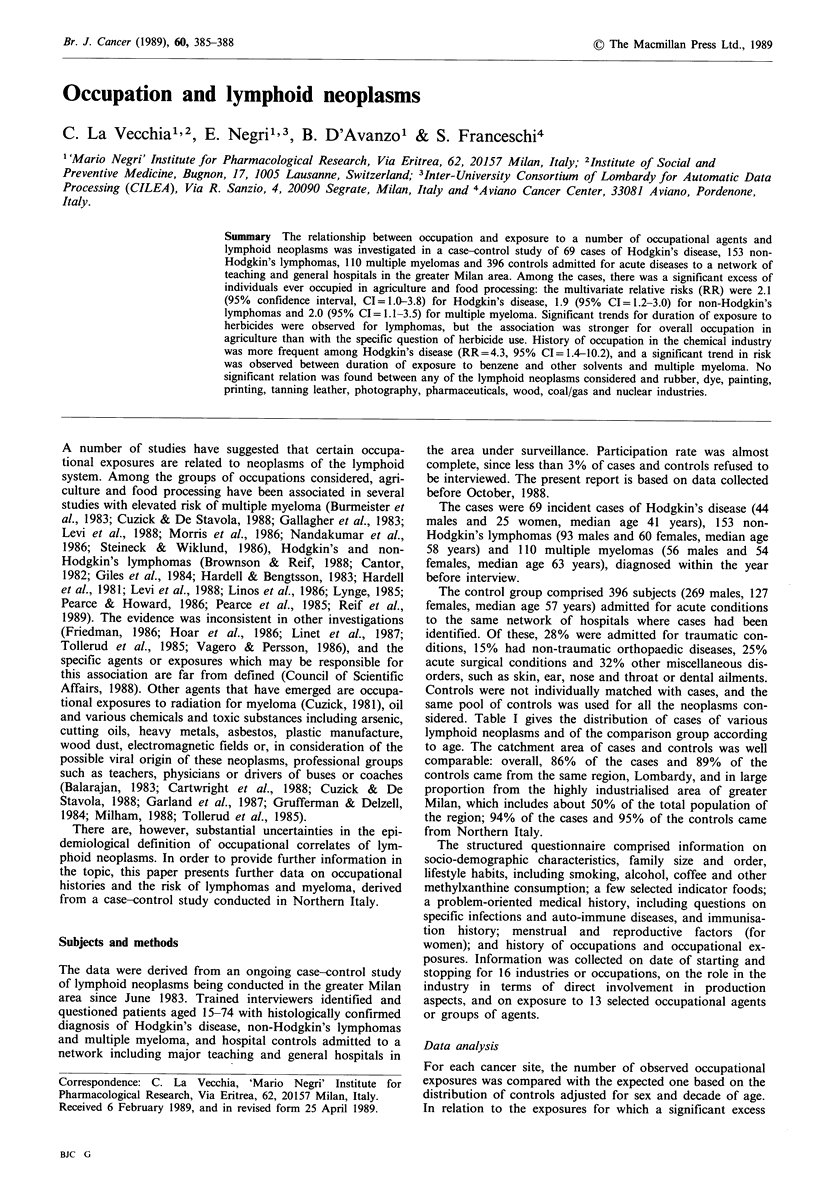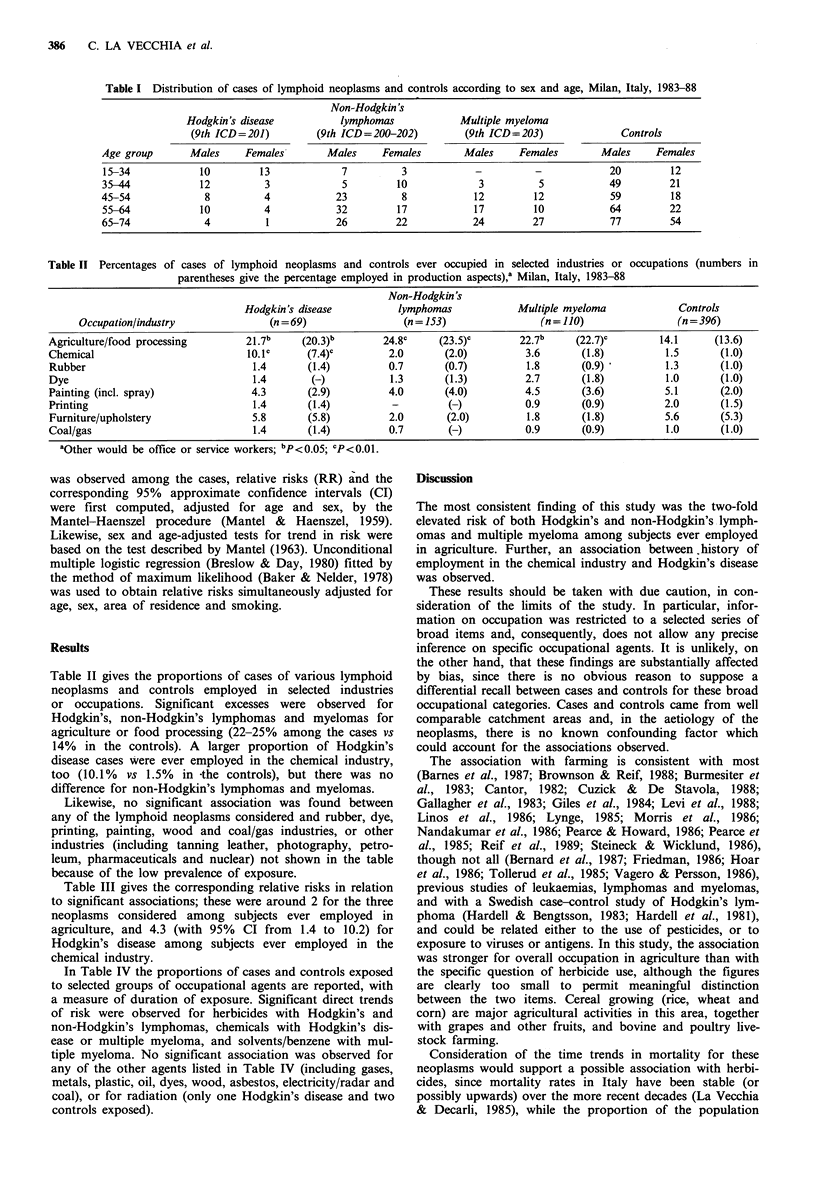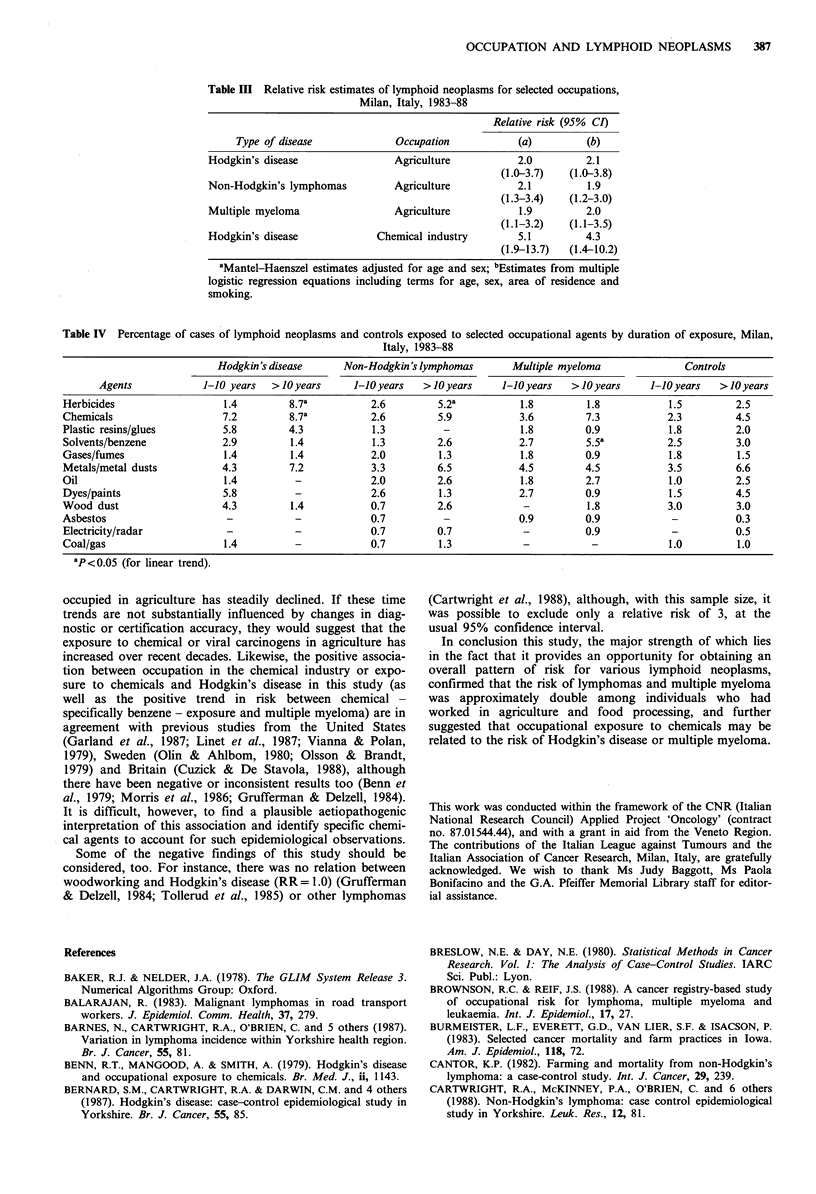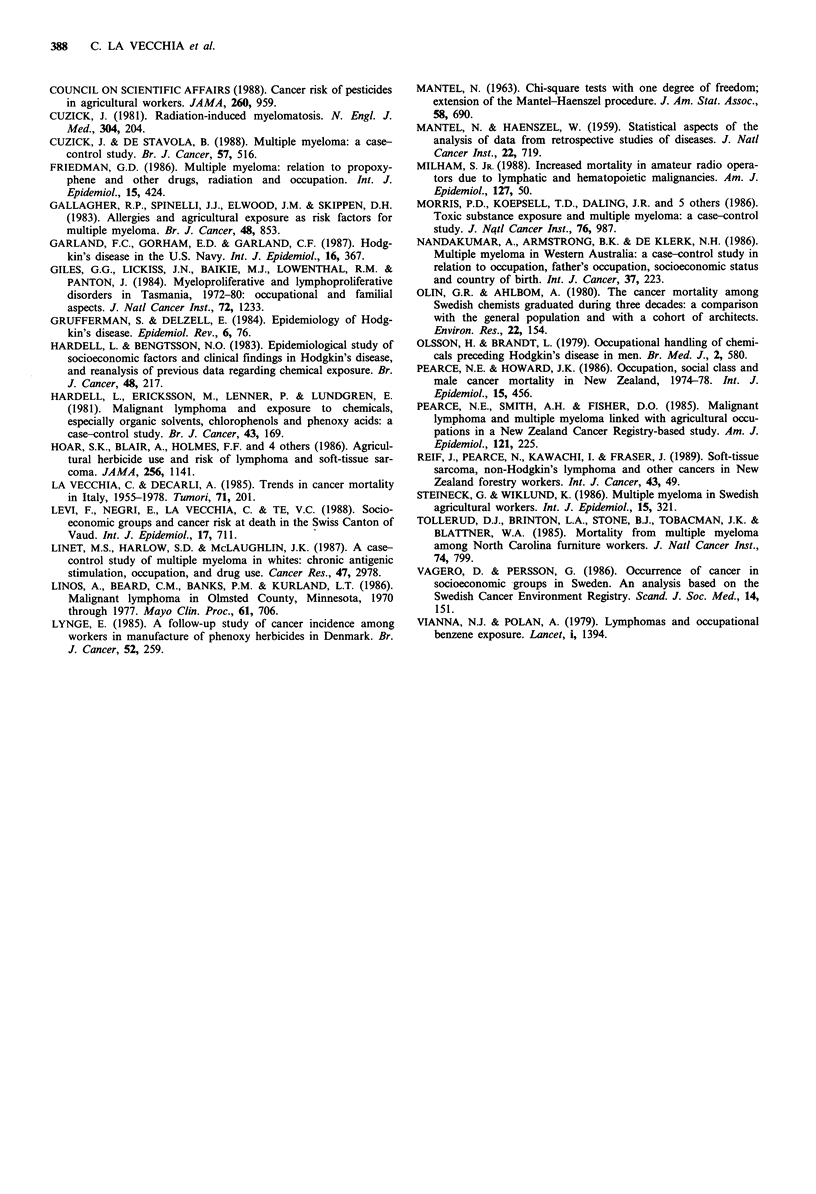# Occupation and lymphoid neoplasms.

**DOI:** 10.1038/bjc.1989.290

**Published:** 1989-09

**Authors:** C. La Vecchia, E. Negri, B. D'Avanzo, S. Franceschi

**Affiliations:** Mario Negri Institute for Pharmacological Research, Milan, Italy.

## Abstract

The relationship between occupation and exposure to a number of occupational agents and lymphoid neoplasms was investigated in a case-control study of 69 cases of Hodgkin's disease, 153 non-Hodgkin's lymphomas, 110 multiple myelomas and 396 controls admitted for acute diseases to a network of teaching and general hospitals in the greater Milan area. Among the cases, there was a significant excess of individuals ever occupied in agriculture and food processing: the multivariate relative risks (RR) were 2.1 (95% confidence interval, CI = 1.0-3.8) for Hodgkin's disease, 1.9 (95% CI = 1.2-3.0) for non-Hodgkin's lymphomas and 2.0 (95% CI = 1.1-3.5) for multiple myeloma. Significant trends for duration of exposure to herbicides were observed for lymphomas, but the association was stronger for overall occupation in agriculture than with the specific question of herbicide use. History of occupation in the chemical industry was more frequent among Hodgkin's disease (RR = 4.3, 95% CI = 1.4-10.2), and a significant trend in risk was observed between duration of exposure to benzene and other solvents and multiple myeloma. No significant relation was found between any of the lymphoid neoplasms considered and rubber, dye, painting, printing, tanning leather, photography, pharmaceuticals, wood, coal/gas and nuclear industries.


*Br. J. Cancer* (1989), **60**, 385–388 © The Macmillan Press Ltd., 1989

### Occupation and lymphoid neoplasms

C. La Vecchia[1,2], E. Negri[1,3], B. D'Avanzo[1] & S. Franceschi[4]

[1]*'Mario Negri' Institute for Pharmacological Research, Via Eritrea, 62, 20157 Milan, Italy;* [2]*Institute of Social and Preventive Medicine, Bugnon, 17, 1005 Lausanne, Switzerland;* [3]*Inter-University Consortium of Lombardy for Automatic Data Processing (CILEA), Via R. Sanzio, 4, 20090 Segrate, Milan, Italy and* [4]*Aviano Cancer Center, 33081 Aviano, Pordenone, Italy.*

**Summary** The relationship between occupation and exposure to a number of occupational agents and lymphoid neoplasms was investigated in a case–control study of 69 cases of Hodgkin's disease, 153 non-Hodgkin's lymphomas, 110 multiple myelomas and 396 controls admitted for acute diseases to a network of teaching and general hospitals in the greater Milan area. Among the cases, there was a significant excess of individuals ever occupied in agriculture and food processing: the multivariate relative risks (RR) were 2.1 (95% confidence interval, CI = 1.0–3.8) for Hodgkin's disease, 1.9 (95% CI = 1.2–3.0) for non-Hodgkin's lymphomas and 2.0 (95% CI = 1.1–3.5) for multiple myeloma. Significant trends for duration of exposure to herbicides were observed for lymphomas, but the association was stronger for overall occupation in agriculture than with the specific question of herbicide use. History of occupation in the chemical industry was more frequent among Hodgkin's disease (RR = 4.3, 95% CI = 1.4–10.2), and a significant trend in risk was observed between duration of exposure to benzene and other solvents and multiple myeloma. No significant relation was found between any of the lymphoid neoplasms considered and rubber, dye, painting, printing, tanning leather, photography, pharmaceuticals, wood, coal/gas and nuclear industries.

A number of studies have suggested that certain occupational exposures are related to neoplasms of the lymphoid system. Among the groups of occupations considered, agriculture and food processing have been associated in several studies with elevated risk of multiple myeloma (Burmeister *et al.*, 1983; Cuzick & De Stavola, 1988; Gallagher *et al.*, 1983; Levi *et al.*, 1988; Morris *et al.*, 1986; Nandakumar *et al.*, 1986; Steineck & Wiklund, 1986), Hodgkin's and non-Hodgkin's lymphomas (Brownson & Reif, 1988; Cantor, 1982; Giles *et al.*, 1984; Hardell & Bengtsson, 1983; Hardell *et al.*, 1981; Levi *et al.*, 1988; Linos *et al.*, 1986; Lynge, 1985; Pearce & Howard, 1986; Pearce *et al.*, 1985; Reif *et al.*, 1989). The evidence was inconsistent in other investigations (Friedman, 1986; Hoar *et al.*, 1986; Linet *et al.*, 1987; Tollerud *et al.*, 1985; Vagero & Persson, 1986), and the specific agents or exposures which may be responsible for this association are far from defined (Council of Scientific Affairs, 1988). Other agents that have emerged are occupational exposures to radiation for myeloma (Cuzick, 1981), oil and various chemicals and toxic substances including arsenic, cutting oils, heavy metals, asbestos, plastic manufacture, wood dust, electromagnetic fields or, in consideration of the possible viral origin of these neoplasms, professional groups such as teachers, physicians or drivers of buses or coaches (Balarajan, 1983; Cartwright *et al.*, 1988; Cuzick & De Stavola, 1988; Garland *et al.*, 1987; Grufferman & Delzell, 1984; Milham, 1988; Tollerud *et al.*, 1985).

There are, however, substantial uncertainties in the epidemiological definition of occupational correlates of lymphoid neoplasms. In order to provide further information in the topic, this paper presents further data on occupational histories and the risk of lymphomas and myeloma, derived from a case–control study conducted in Northern Italy.

### Subjects and methods

The data were derived from an ongoing case–control study of lymphoid neoplasms being conducted in the greater Milan area since June 1983. Trained interviewers identified and questioned patients aged 15–74 with histologically confirmed diagnosis of Hodgkin's disease, non-Hodgkin's lymphomas and multiple myeloma, and hospital controls admitted to a network including major teaching and general hospitals in the area under surveillance. Participation rate was almost complete, since less than 3% of cases and controls refused to be interviewed. The present report is based on data collected before October, 1988.

The cases were 69 incident cases of Hodgkin's disease (44 males and 25 women, median age 41 years), 153 non-Hodgkin's lymphomas (93 males and 60 females, median age 58 years) and 110 multiple myelomas (56 males and 54 females, median age 63 years), diagnosed within the year before interview.

The control group comprised 396 subjects (269 males, 127 females, median age 57 years) admitted for acute conditions to the same network of hospitals where cases had been identified. Of these, 28% were admitted for traumatic conditions, 15% had non-traumatic orthopaedic diseases, 25% acute surgical conditions and 32% other miscellaneous disorders, such as skin, ear, nose and throat or dental ailments. Controls were not individually matched with cases, and the same pool of controls was used for all the neoplasms considered. Table I gives the distribution of cases of various lymphoid neoplasms and of the comparison group according to age. The catchment area of cases and controls was well comparable: overall, 86% of the cases and 89% of the controls came from the same region, Lombardy, and in large proportion from the highly industrialised area of greater Milan, which includes about 50% of the total population of the region; 94% of the cases and 95% of the controls came from Northern Italy.

The structured questionnaire comprised information on socio-demographic characteristics, family size and order, lifestyle habits, including smoking, alcohol, coffee and other methylxanthine consumption; a few selected indicator foods; a problem-oriented medical history, including questions on specific infections and auto-immune diseases, and immunisation history; menstrual and reproductive factors (for women); and history of occupations and occupational exposures. Information was collected on date of starting and stopping for 16 industries or occupations, on the role in the industry in terms of direct involvement in production aspects, and on exposure to 13 selected occupational agents or groups of agents.

### *Data analysis*

For each cancer site, the number of observed occupational exposures was compared with the expected one based on the distribution of controls adjusted for sex and decade of age. In relation to the exposures for which a significant excess

Correspondence: C. La Vecchia, 'Mario Negri' Institute for Pharmacological Research, Via Eritrea, 62, 20157 Milan, Italy.
Received 6 February 1989, and in revised form 25 April 1989.

**Table I** Distribution of cases of lymphoid neoplasms and controls according to sex and age, Milan, Italy, 1983–88

| | *Hodgkin's disease (9th ICD=201)* | | *Non-Hodgkin's lymphomas (9th ICD=200–202)* | | *Multiple myeloma (9th ICD=203)* | | *Controls* | |
|---|---|---|---|---|---|---|---|---|
| *Age group* | *Males* | *Females* | *Males* | *Females* | *Males* | *Females* | *Males* | *Females* |
| 15–34 | 10 | 13 | 7 | 3 | – | – | 20 | 12 |
| 35–44 | 12 | 3 | 5 | 10 | 3 | 5 | 49 | 21 |
| 45–54 | 8 | 4 | 23 | 8 | 12 | 12 | 59 | 18 |
| 55–64 | 10 | 4 | 32 | 17 | 17 | 10 | 64 | 22 |
| 65–74 | 4 | 1 | 26 | 22 | 24 | 27 | 77 | 54 |

**Table II** Percentages of cases of lymphoid neoplasms and controls ever occupied in selected industries or occupations (numbers in parentheses give the percentage employed in production aspects),[a] Milan, Italy, 1983–88

| *Occupation/industry* | *Hodgkin's disease (n=69)* | | *Non-Hodgkin's lymphomas (n=153)* | | *Multiple myeloma (n=110)* | | *Controls (n=396)* | |
|---|---|---|---|---|---|---|---|---|
| Agriculture/food processing | 21.7[b] | (20.3)[b] | 24.8[c] | (23.5)[c] | 22.7[b] | (22.7)[c] | 14.1 | (13.6) |
| Chemical | 10.1[c] | (7.4)[c] | 2.0 | (2.0) | 3.6 | (1.8) | 1.5 | (1.0) |
| Rubber | 1.4 | (1.4) | 0.7 | (0.7) | 1.8 | (0.9) | 1.3 | (1.0) |
| Dye | 1.4 | (–) | 1.3 | (1.3) | 2.7 | (1.8) | 1.0 | (1.0) |
| Painting (incl. spray) | 4.3 | (2.9) | 4.0 | (4.0) | 4.5 | (3.6) | 5.1 | (2.0) |
| Printing | 1.4 | (1.4) | – | (–) | 0.9 | (0.9) | 2.0 | (1.5) |
| Furniture/upholstery | 5.8 | (5.8) | 2.0 | (2.0) | 1.8 | (1.8) | 5.6 | (5.3) |
| Coal/gas | 1.4 | (1.4) | 0.7 | (–) | 0.9 | (0.9) | 1.0 | (1.0) |

[a]Other would be office or service workers; [b]$P<0.05$; [c]$P<0.01$.

was observed among the cases, relative risks (RR) and the corresponding 95% approximate confidence intervals (CI) were first computed, adjusted for age and sex, by the Mantel–Haenszel procedure (Mantel & Haenszel, 1959). Likewise, sex and age-adjusted tests for trend in risk were based on the test described by Mantel (1963). Unconditional multiple logistic regression (Breslow & Day, 1980) fitted by the method of maximum likelihood (Baker & Nelder, 1978) was used to obtain relative risks simultaneously adjusted for age, sex, area of residence and smoking.

### Results

Table II gives the proportions of cases of various lymphoid neoplasms and controls employed in selected industries or occupations. Significant excesses were observed for Hodgkin's, non-Hodgkin's lymphomas and myelomas for agriculture or food processing (22–25% among the cases *vs* 14% in the controls). A larger proportion of Hodgkin's disease cases were ever employed in the chemical industry, too (10.1% *vs* 1.5% in the controls), but there was no difference for non-Hodgkin's lymphomas and myelomas.

Likewise, no significant association was found between any of the lymphoid neoplasms considered and rubber, dye, printing, painting, wood and coal/gas industries, or other industries (including tanning leather, photography, petroleum, pharmaceuticals and nuclear) not shown in the table because of the low prevalence of exposure.

Table III gives the corresponding relative risks in relation to significant associations; these were around 2 for the three neoplasms considered among subjects ever employed in agriculture, and 4.3 (with 95% CI from 1.4 to 10.2) for Hodgkin's disease among subjects ever employed in the chemical industry.

In Table IV the proportions of cases and controls exposed to selected groups of occupational agents are reported, with a measure of duration of exposure. Significant direct trends of risk were observed for herbicides with Hodgkin's and non-Hodgkin's lymphomas, chemicals with Hodgkin's disease or multiple myeloma, and solvents/benzene with multiple myeloma. No significant association was observed for any of the other agents listed in Table IV (including gases, metals, plastic, oil, dyes, wood, asbestos, electricity/radar and coal), or for radiation (only one Hodgkin's disease and two controls exposed).

### Discussion

The most consistent finding of this study was the two-fold elevated risk of both Hodgkin's and non-Hodgkin's lymphomas and multiple myeloma among subjects ever employed in agriculture. Further, an association between history of employment in the chemical industry and Hodgkin's disease was observed.

These results should be taken with due caution, in consideration of the limits of the study. In particular, information on occupation was restricted to a selected series of broad items and, consequently, does not allow any precise inference on specific occupational agents. It is unlikely, on the other hand, that these findings are substantially affected by bias, since there is no obvious reason to suppose a differential recall between cases and controls for these broad occupational categories. Cases and controls came from well comparable catchment areas and, in the aetiology of the neoplasms, there is no known confounding factor which could account for the associations observed.

The association with farming is consistent with most (Barnes *et al.*, 1987; Brownson & Reif, 1988; Burmesiter *et al.*, 1983; Cantor, 1982; Cuzick & De Stavola, 1988; Gallagher *et al.*, 1983; Giles *et al.*, 1984; Levi *et al.*, 1988; Linos *et al.*, 1986; Lynge, 1985; Morris *et al.*, 1986; Nandakumar *et al.*, 1986; Pearce & Howard, 1986; Pearce *et al.*, 1985; Reif *et al.*, 1989; Steineck & Wicklund, 1986), though not all (Bernard *et al.*, 1987; Friedman, 1986; Hoar *et al.*, 1986; Tollerud *et al.*, 1985; Vagero & Persson, 1986), previous studies of leukaemias, lymphomas and myelomas, and with a Swedish case–control study of Hodgkin's lymphoma (Hardell & Bengtsson, 1983; Hardell *et al.*, 1981), and could be related either to the use of pesticides, or to exposure to viruses or antigens. In this study, the association was stronger for overall occupation in agriculture than with the specific question of herbicide use, although the figures are clearly too small to permit meaningful distinction between the two items. Cereal growing (rice, wheat and corn) are major agricultural activities in this area, together with grapes and other fruits, and bovine and poultry livestock farming.

Consideration of the time trends in mortality for these neoplasms would support a possible association with herbicides, since mortality rates in Italy have been stable (or possibly upwards) over the more recent decades (La Vecchia & Decarli, 1985), while the proportion of the population

**Table III** Relative risk estimates of lymphoid neoplasms for selected occupations, Milan, Italy, 1983–88

| Type of disease | Occupation | Relative risk (95% CI) (a) | (b) |
|---|---|---|---|
| Hodgkin's disease | Agriculture | 2.0 (1.0–3.7) | 2.1 (1.0–3.8) |
| Non-Hodgkin's lymphomas | Agriculture | 2.1 (1.3–3.4) | 1.9 (1.2–3.0) |
| Multiple myeloma | Agriculture | 1.9 (1.1–3.2) | 2.0 (1.1–3.5) |
| Hodgkin's disease | Chemical industry | 5.1 (1.9–13.7) | 4.3 (1.4–10.2) |

[a]Mantel–Haenszel estimates adjusted for age and sex; [b]Estimates from multiple logistic regression equations including terms for age, sex, area of residence and smoking.

**Table IV** Percentage of cases of lymphoid neoplasms and controls exposed to selected occupational agents by duration of exposure, Milan, Italy, 1983–88

| Agents | Hodgkin's disease 1–10 years | Hodgkin's disease >10 years | Non-Hodgkin's lymphomas 1–10 years | Non-Hodgkin's lymphomas >10 years | Multiple myeloma 1–10 years | Multiple myeloma >10 years | Controls 1–10 years | Controls >10 years |
|---|---|---|---|---|---|---|---|---|
| Herbicides | 1.4 | 8.7[a] | 2.6 | 5.2[a] | 1.8 | 1.8 | 1.5 | 2.5 |
| Chemicals | 7.2 | 8.7[a] | 2.6 | 5.9 | 3.6 | 7.3 | 2.3 | 4.5 |
| Plastic resins/glues | 5.8 | 4.3 | 1.3 | – | 1.8 | 0.9 | 1.8 | 2.0 |
| Solvents/benzene | 2.9 | 1.4 | 1.3 | 2.6 | 2.7 | 5.5[a] | 2.5 | 3.0 |
| Gases/fumes | 1.4 | 1.4 | 2.0 | 1.3 | 1.8 | 0.9 | 1.8 | 1.5 |
| Metals/metal dusts | 4.3 | 7.2 | 3.3 | 6.5 | 4.5 | 4.5 | 3.5 | 6.6 |
| Oil | 1.4 | – | 2.0 | 2.6 | 1.8 | 2.7 | 1.0 | 2.5 |
| Dyes/paints | 5.8 | – | 2.6 | 1.3 | 2.7 | 0.9 | 1.5 | 4.5 |
| Wood dust | 4.3 | 1.4 | 0.7 | 2.6 | – | 1.8 | 3.0 | 3.0 |
| Asbestos | – | – | 0.7 | – | 0.9 | 0.9 | – | 0.3 |
| Electricity/radar | – | – | 0.7 | 0.7 | – | 0.9 | – | 0.5 |
| Coal/gas | 1.4 | – | 0.7 | 1.3 | – | – | 1.0 | 1.0 |

[a]$P<0.05$ (for linear trend).

occupied in agriculture has steadily declined. If these time trends are not substantially influenced by changes in diagnostic or certification accuracy, they would suggest that the exposure to chemical or viral carcinogens in agriculture has increased over recent decades. Likewise, the positive association between occupation in the chemical industry or exposure to chemicals and Hodgkin's disease in this study (as well as the positive trend in risk between chemical – specifically benzene – exposure and multiple myeloma) are in agreement with previous studies from the United States (Garland *et al.*, 1987; Linet *et al.*, 1987; Vianna & Polan, 1979), Sweden (Olin & Ahlbom, 1980; Olsson & Brandt, 1979) and Britain (Cuzick & De Stavola, 1988), although there have been negative or inconsistent results too (Benn *et al.*, 1979; Morris *et al.*, 1986; Grufferman & Delzell, 1984). It is difficult, however, to find a plausible aetiopathogenic interpretation of this association and identify specific chemical agents to account for such epidemiological observations.

Some of the negative findings of this study should be considered, too. For instance, there was no relation between woodworking and Hodgkin's disease (RR = 1.0) (Grufferman & Delzell, 1984; Tollerud *et al.*, 1985) or other lymphomas (Cartwright *et al.*, 1988), although, with this sample size, it was possible to exclude only a relative risk of 3, at the usual 95% confidence interval.

In conclusion this study, the major strength of which lies in the fact that it provides an opportunity for obtaining an overall pattern of risk for various lymphoid neoplasms, confirmed that the risk of lymphomas and multiple myeloma was approximately double among individuals who had worked in agriculture and food processing, and further suggested that occupational exposure to chemicals may be related to the risk of Hodgkin's disease or multiple myeloma.

This work was conducted within the framework of the CNR (Italian National Research Council) Applied Project 'Oncology' (contract no. 87.01544.44), and with a grant in aid from the Veneto Region. The contributions of the Italian League against Tumours and the Italian Association of Cancer Research, Milan, Italy, are gratefully acknowledged. We wish to thank Ms Judy Baggott, Ms Paola Bonifacino and the G.A. Pfeiffer Memorial Library staff for editorial assistance.

### References

BAKER, R.J. & NELDER, J.A. (1978). *The GLIM System Release 3.* Numerical Algorithms Group: Oxford.

BALARAJAN, R. (1983). Malignant lymphomas in road transport workers. *J. Epidemiol. Comm. Health*, **37**, 279.

BARNES, N., CARTWRIGHT, R.A., O'BRIEN, C. and 5 others (1987). Variation in lymphoma incidence within Yorkshire health region. *Br. J. Cancer*, **55**, 81.

BENN, R.T., MANGOOD, A. & SMITH, A. (1979). Hodgkin's disease and occupational exposure to chemicals. *Br. Med. J.*, **ii**, 1143.

BERNARD, S.M., CARTWRIGHT, R.A. & DARWIN, C.M. and 4 others (1987). Hodgkin's disease: case–control epidemiological study in Yorkshire. *Br. J. Cancer*, **55**, 85.

BRESLOW, N.E. & DAY, N.E. (1980). *Statistical Methods in Cancer Research. Vol. 1: The Analysis of Case–Control Studies.* IARC Sci. Publ.: Lyon.

BROWNSON, R.C. & REIF, J.S. (1988). A cancer registry-based study of occupational risk for lymphoma, multiple myeloma and leukaemia. *Int. J. Epidemiol.*, **17**, 27.

BURMEISTER, L.F., EVERETT, G.D., VAN LIER, S.F. & ISACSON, P. (1983). Selected cancer mortality and farm practices in Iowa. *Am. J. Epidemiol.*, **118**, 72.

CANTOR, K.P. (1982). Farming and mortality from non-Hodgkin's lymphoma: a case-control study. *Int. J. Cancer*, **29**, 239.

CARTWRIGHT, R.A., McKINNEY, P.A., O'BRIEN, C. and 6 others (1988). Non-Hodgkin's lymphoma: case control epidemiological study in Yorkshire. *Leuk. Res.*, **12**, 81.

COUNCIL ON SCIENTIFIC AFFAIRS (1988). Cancer risk of pesticides in agricultural workers. *JAMA*, **260**, 959.
CUZICK, J. (1981). Radiation-induced myelomatosis. *N. Engl. J. Med.*, **304**, 204.
CUZICK, J. & DE STAVOLA, B. (1988). Multiple myeloma: a case–control study. *Br. J. Cancer*, **57**, 516.
FRIEDMAN, G.D. (1986). Multiple myeloma: relation to propoxyphene and other drugs, radiation and occupation. *Int. J. Epidemiol.*, **15**, 424.
GALLAGHER, R.P., SPINELLI, J.J., ELWOOD, J.M. & SKIPPEN, D.H. (1983). Allergies and agricultural exposure as risk factors for multiple myeloma. *Br. J. Cancer*, **48**, 853.
GARLAND, F.C., GORHAM, E.D. & GARLAND, C.F. (1987). Hodgkin's disease in the U.S. Navy. *Int. J. Epidemiol.*, **16**, 367.
GILES, G.G., LICKISS, J.N., BAIKIE, M.J., LOWENTHAL, R.M. & PANTON, J. (1984). Myeloproliferative and lymphoproliferative disorders in Tasmania, 1972–80: occupational and familial aspects. *J. Natl Cancer Inst.*, **72**, 1233.
GRUFFERMAN, S. & DELZELL, E. (1984). Epidemiology of Hodgkin's disease. *Epidemiol. Rev.*, **6**, 76.
HARDELL, L. & BENGTSSON, N.O. (1983). Epidemiological study of socioeconomic factors and clinical findings in Hodgkin's disease, and reanalysis of previous data regarding chemical exposure. *Br. J. Cancer*, **48**, 217.
HARDELL, L., ERICKSSON, M., LENNER, P. & LUNDGREN, E. (1981). Malignant lymphoma and exposure to chemicals, especially organic solvents, chlorophenols and phenoxy acids: a case–control study. *Br. J. Cancer*, **43**, 169.
HOAR, S.K., BLAIR, A., HOLMES, F.F. and 4 others (1986). Agricultural herbicide use and risk of lymphoma and soft-tissue sarcoma. *JAMA*, **256**, 1141.
LA VECCHIA, C. & DECARLI, A. (1985). Trends in cancer mortality in Italy, 1955–1978. *Tumori*, **71**, 201.
LEVI, F., NEGRI, E., LA VECCHIA, C. & TE, V.C. (1988). Socioeconomic groups and cancer risk at death in the Swiss Canton of Vaud. *Int. J. Epidemiol.*, **17**, 711.
LINET, M.S., HARLOW, S.D. & McLAUGHLIN, J.K. (1987). A case–control study of multiple myeloma in whites: chronic antigenic stimulation, occupation, and drug use. *Cancer Res.*, **47**, 2978.
LINOS, A., BEARD, C.M., BANKS, P.M. & KURLAND, L.T. (1986). Malignant lymphoma in Olmsted County, Minnesota, 1970 through 1977. *Mayo Clin. Proc.*, **61**, 706.
LYNGE, E. (1985). A follow-up study of cancer incidence among workers in manufacture of phenoxy herbicides in Denmark. *Br. J. Cancer*, **52**, 259.
MANTEL, N. (1963). Chi-square tests with one degree of freedom; extension of the Mantel–Haenszel procedure. *J. Am. Stat. Assoc.*, **58**, 690.
MANTEL, N. & HAENSZEL, W. (1959). Statistical aspects of the analysis of data from retrospective studies of diseases. *J. Natl Cancer Inst.*, **22**, 719.
MILHAM, S. JR. (1988). Increased mortality in amateur radio operators due to lymphatic and hematopoietic malignancies. *Am. J. Epidemiol.*, **127**, 50.
MORRIS, P.D., KOEPSELL, T.D., DALING, J.R. and 5 others (1986). Toxic substance exposure and multiple myeloma: a case–control study. *J. Natl Cancer Inst.*, **76**, 987.
NANDAKUMAR, A., ARMSTRONG, B.K. & DE KLERK, N.H. (1986). Multiple myeloma in Western Australia: a case–control study in relation to occupation, father's occupation, socioeconomic status and country of birth. *Int. J. Cancer*, **37**, 223.
OLIN, G.R. & AHLBOM, A. (1980). The cancer mortality among Swedish chemists graduated during three decades: a comparison with the general population and with a cohort of architects. *Environ. Res.*, **22**, 154.
OLSSON, H. & BRANDT, L. (1979). Occupational handling of chemicals preceding Hodgkin's disease in men. *Br. Med. J.*, **2**, 580.
PEARCE, N.E. & HOWARD, J.K. (1986). Occupation, social class and male cancer mortality in New Zealand, 1974–78. *Int. J. Epidemiol.*, **15**, 456.
PEARCE, N.E., SMITH, A.H. & FISHER, D.O. (1985). Malignant lymphoma and multiple myeloma linked with agricultural occupations in a New Zealand Cancer Registry-based study. *Am. J. Epidemiol.*, **121**, 225.
REIF, J., PEARCE, N., KAWACHI, I. & FRASER, J. (1989). Soft-tissue sarcoma, non-Hodgkin's lymphoma and other cancers in New Zealand forestry workers. *Int. J. Cancer*, **43**, 49.
STEINECK, G. & WIKLUND, K. (1986). Multiple myeloma in Swedish agricultural workers. *Int. J. Epidemiol.*, **15**, 321.
TOLLERUD, D.J., BRINTON, L.A., STONE, B.J., TOBACMAN, J.K. & BLATTNER, W.A. (1985). Mortality from multiple myeloma among North Carolina furniture workers. *J. Natl Cancer Inst.*, **74**, 799.
VAGERO, D. & PERSSON, G. (1986). Occurrence of cancer in socioeconomic groups in Sweden. An analysis based on the Swedish Cancer Environment Registry. *Scand. J. Soc. Med.*, **14**, 151.
VIANNA, N.J. & POLAN, A. (1979). Lymphomas and occupational benzene exposure. *Lancet*, **i**, 1394.